# 164. Effectiveness of Adjuvanted Inactivated Influenza Vaccine versus High-Dose Inactivated Influenza Vaccine Against PCR-Confirmed Influenza among Adults ≥65 years: A Pragmatic Randomized Study

**DOI:** 10.1093/ofid/ofae631.001

**Published:** 2025-01-29

**Authors:** Amber Hsiao, Arnold Yee, Thomas Leong, Ousseny Zerbo, Bruce Fireman, Karen B Jacobson, John R Hansen, Evan Layefsky, Mendel Haag, Ian McGovern, Bin Zhang, Juliet Dang, Nicola P Klein

**Affiliations:** Division of Research Kaiser Permanente Vaccine Study Center, Oakland, California; Division of Research Kaiser Permanente Vaccine Study Center, Oakland, California; Kaiser Permanente Northern California, Oakland, California; Division of Research Kaiser Permanente Vaccine Study Center, Oakland, California; Division of Research Kaiser Permanente Vaccine Study Center, Oakland, California; Kaiser Permanente Vaccine Study Center, Oakland, California; Division of Research Kaiser Permanente Vaccine Study Center, Oakland, California; Division of Research Kaiser Permanente Vaccine Study Center, Oakland, California; CSL Seqirus, Amsterdam, Noord-Holland, Netherlands; CSL Seqirus, Amsterdam, Noord-Holland, Netherlands; CSL Seqirus, Amsterdam, Noord-Holland, Netherlands; CSL Seqirus, Amsterdam, Noord-Holland, Netherlands; Division of Research Kaiser Permanente Vaccine Study Center, Oakland, California

## Abstract

**Background:**

In the US, adults aged ≥65 years are recommended to receive an adjuvanted or higher dose influenza vaccine. Adjuvanted (aIIV) and high-dose (HD-IIV) inactivated influenza vaccines were similarly effective against influenza in several studies based on diagnostic codes. However, less is known about their relative vaccine effectiveness (rVE) against laboratory-confirmed influenza and they have never been compared in a randomized study. We assessed the rVE of adjuvanted vs. high-dose vaccines against PCR-confirmed influenza in adults aged ≥65 years at Kaiser Permanente Northern California (KPNC).

**Figure:**
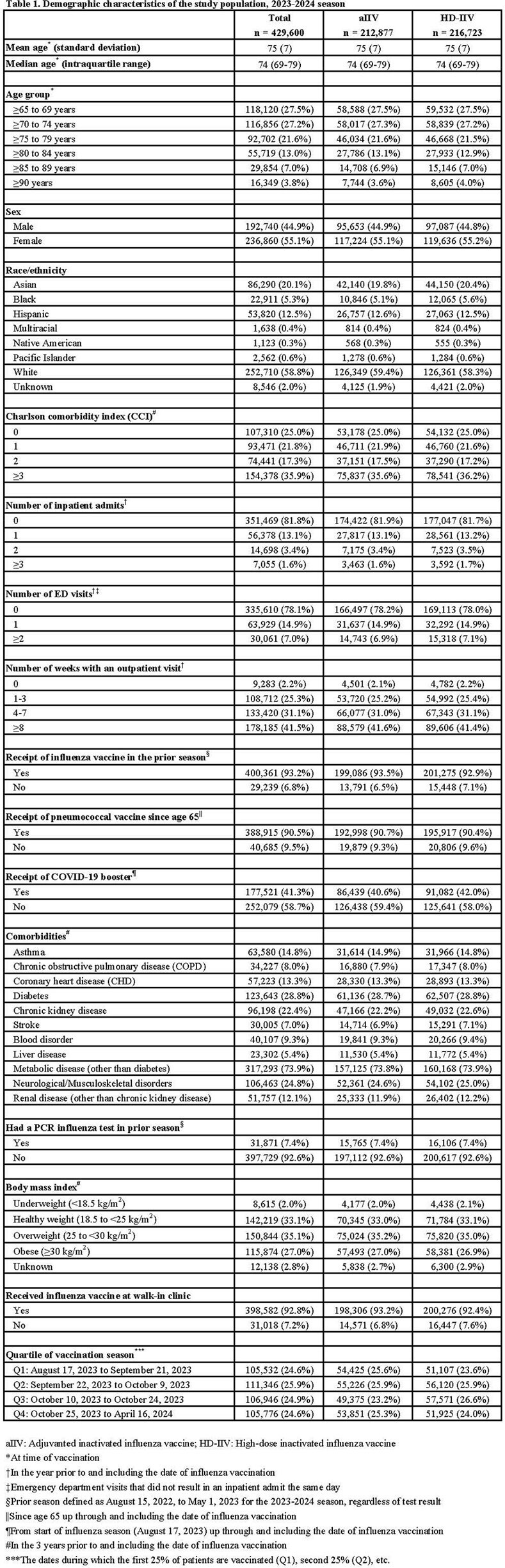

**Methods:**

During the 2023-2024 influenza season, we randomized all KPNC facilities to administer aIIV vs. HD-IIV in alternating weeks to achieve balance between the two vaccine groups. Individuals received either aIIV or HD-IIV depending on which facility and week they sought vaccination. The primary outcome was PCR-confirmed influenza in any clinical setting. Secondary outcomes were hospitalizations or emergency department (ED) visits for PCR-confirmed influenza and hospitalizations for community-acquired pneumonia (CAP). Using Cox regression on a calendar timeline, we estimated the relative vaccine effectiveness (rVE) of aIIV vs. HD-IIV as 1 minus the adjusted hazard ratio, adjusted for age, sex, race/ethnicity, and other covariates.

**Figure:**
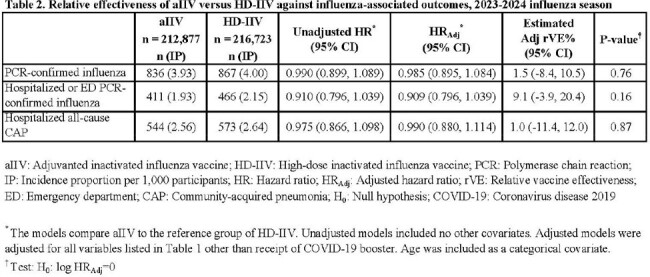

**Results:**

The study population included 429,600 individuals; 212,887 (49.6%) received aIIV and 216,723 (50.4%) received HD-IIV. Baseline characteristics were well balanced between the two groups (Table 1). There were 836 cases of PCR-confirmed influenza (3.9 per 1000 persons) after aIIV and 867 cases (4.0 per 1000 persons) after HD-IIV. Comparing aIIV with HD-IIV, the rVE against PCR-confirmed influenza was 1.5% (95% CI: -8.4%, 10.5%; Table 2). AIIV and HD-IIV also did not differ in effectiveness against PCR-confirmed influenza hospitalizations/ED visits (rVE: 9.1%; CI: -3.9%, 20.4%) and CAP hospitalizations (rVE: 1.0%; CI: -11.4%, 12.0%).

**Conclusion:**

In this large, pragmatic randomized study in adults aged ≥65 years during the 2023-2024 influenza season, adjuvanted and high-dose influenza vaccines did not differ in effectiveness against laboratory-confirmed influenza.

**Disclosures:**

**Mendel Haag, PhD, PharmD**, CSL Seqirus: Employee|CSL Seqirus: Stocks/Bonds (Public Company) **Ian McGovern, MPH**, CSL Seqirus: employee|CSL Seqirus: Stocks/Bonds (Public Company) **Bin Zhang, ScD, MA**, CSL Seqirus: Employee|CSL Seqirus: Stocks/Bonds (Public Company) **Juliet Dang, PhD, MS**, CSL Seqirus: Employee|CSL Seqirus: Stocks/Bonds (Public Company) **Nicola P. Klein, MD, PhD**, GlaxoSmithKline: Grant/Research Support|Merck: Grant/Research Support|Pfizer: Grant/Research Support|Sanofi Pasteur: Grant/Research Support|Seqirus: Grant/Research Support

